# Isolated Right Hepatic Duct Biliary Cyst With a Literature Review: Is It Time to Expand the Todani Classification?

**DOI:** 10.7759/cureus.88252

**Published:** 2025-07-18

**Authors:** Priya Gupta, Roysuneel Patankar, Surendra K Mathur

**Affiliations:** 1 Department of Digestive Diseases, Zen Multispeciality Hospital, Mumbai, IND; 2 Department of Surgery, Zen Multispeciality Hospital, Mumbai, IND; 3 Department of Gastrosurgery, Zen Multispeciality Hospital, Mumbai, IND

**Keywords:** biliary duct cyst, common hepatic duct diverticulum, intrahepatic duct cyst, recurrent cholangitis, todani classification

## Abstract

Biliary tract cysts are congenital intrahepatic and/or extrahepatic cystic dilatations of the biliary system. Todani classified the biliary tract cysts into five categories. However, solitary intrahepatic biliary tract cysts are rare. With the advancement of better imaging modalities over the decades, new cases have been reported that do not fit into the original Todani classification of biliary duct cysts. Hence, we propose an expansion of the Todani classification to include the uncommon variants.

## Introduction

Biliary tract cysts are benign congenital cystic dilatations of the biliary system and can be intrahepatic and/or extrahepatic. Most commonly diagnosed in infancy and childhood, about 20-25% of cases are recorded in adults [1]. They are an important clinical entity requiring correct identification and timely management. Clinical presentation and treatment depend upon the type of biliary cyst. Complications of biliary cysts include cholelithiasis and hepatolithiasis, cholangitis, acute and chronic pancreatitis, portal hypertension, liver fibrosis, secondary liver cirrhosis, and spontaneous cyst perforation. Cholangiocarcinoma is the most serious complication [2]. In 1977, Todani classified biliary tract cysts into five categories [3], where intrahepatic bile duct cysts (single or multiple) were classified as type V. Caroli’s disease, defined as segmental dilatation of multiple, large, intrahepatic bile ducts, without hepatic fibrosis, is a classic example of type V biliary tract cysts. However, solitary intrahepatic biliary tract cysts are extremely rare. Here, we describe the case of a young adult female patient who presented with repeated episodes of cholangitis due to a solitary biliary tract cyst in the right hepatic duct, which has been now termed as type Va. Advancements in imaging modalities have identified cases that do not fit the original Todani classification of biliary cysts, necessitating an expansion of the existing classification. Thus, we propose an expansion of the Todani classification to include these uncommon variants: type VA, VB, VIA, VIB, and VII cysts.

## Case presentation

A 25-year-old female presented to the outpatient department with complaints of recurrent attacks of fever with chills and epigastric pain. There was no history of jaundice, urinary complaints, or similar episodes. On presentation, she had low-grade fever (99°F) and tachycardia (pulse, 102 beats/minute). However, there was no icterus. On per abdomen examination, mild tenderness was present in the epigastric region. Her blood investigations showed leukocytosis and elevated liver parameters (Table 1).

**Table 1:** Blood investigations. AST: aspartate aminotransferase; ALT: alanine aminotransferase; GGT: gamma-glutamyltransferase; ALP: alkaline phosphatase

Parameter	Patient values	Normal range
Total leucocyte count	16,000	4,000–11,000/μL
Total bilirubin	1.55	0.1–0.9 mg/dL
Direct bilirubin	1.43	0.1–0.3 mg/dL
AST	136.1	5–40 IU/L
ALT	91.1	5–40 IU/L
GGT	273.8	5–40 IU/L
ALP	158.4	45–145 U/L

**Figure 1:**
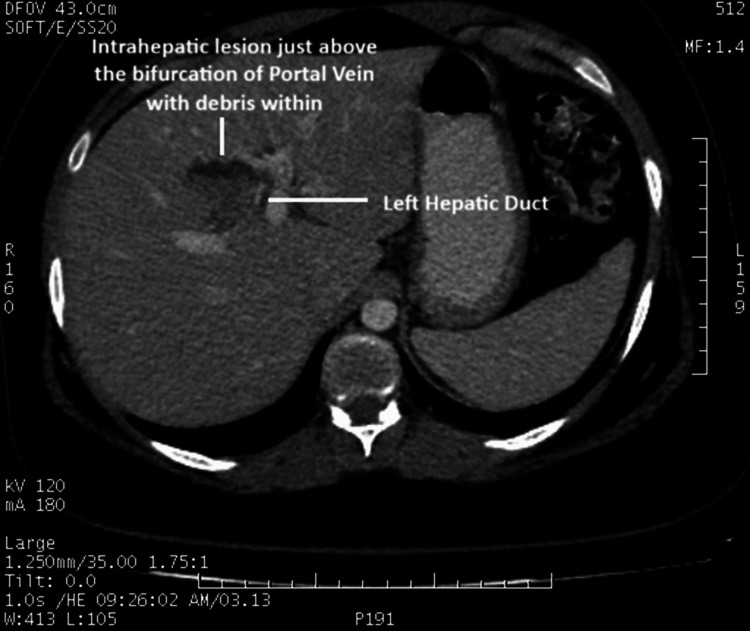
Axial CT scan showing the intrahepatic lesion with debris.

Ultrasonography (USG) of the abdomen revealed an ill-defined anechoic lesion with echogenic debris within segment IV of the liver, suggestive of a liver abscess. A contrast-enhanced CT scan of the abdomen was suggestive of an ill-defined, hypodense, hypoenhancing lesion in the liver just above the bifurcation of the portal vein with debris within, without intra- or extrahepatic biliary dilatation (Figure 1).

**Figure 2:**
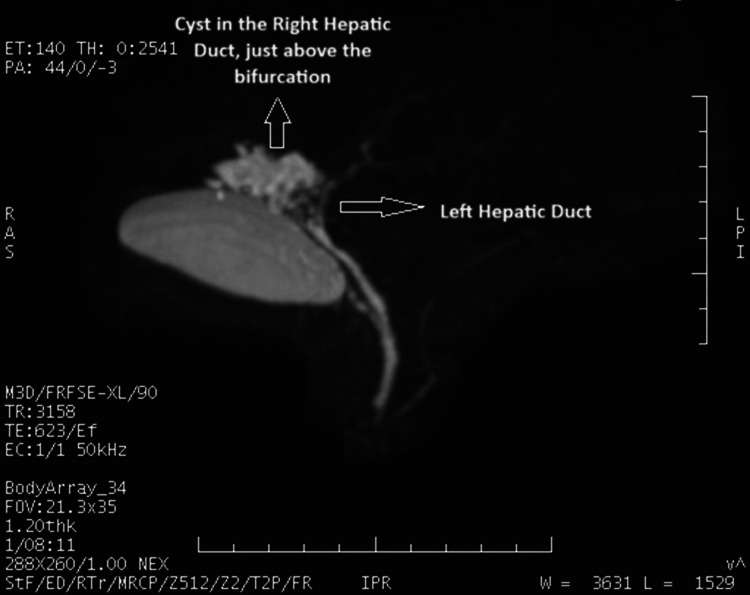
magnetic resonance cholangiopancreatography with a cystic lesion in the right hepatic duct with multiple caculi.

With a diagnosis of evolving liver abscess, the patient was treated with a two-week course of antibiotics and metronidazole. However, the patient presented again with fever after 10 days. Repeat USG of the abdomen revealed multiple, immobile, echogenic areas along the region of the cystic duct, suggestive of the possibility of intrahepatic biliary calculi. Hence, magnetic resonance cholangiopancreatography was done, which showed a well-defined, lobulated cystic lesion (measuring about 35 × 33 × 30 mm) in the periportal region in segment IV of the liver, involving the proximal right hepatic duct near the confluence, with multiple filling defects with a possibility of a biliary diverticulum or choledochal cyst with multiple small secondary calculi (Figure 2).

**Figure 3:**
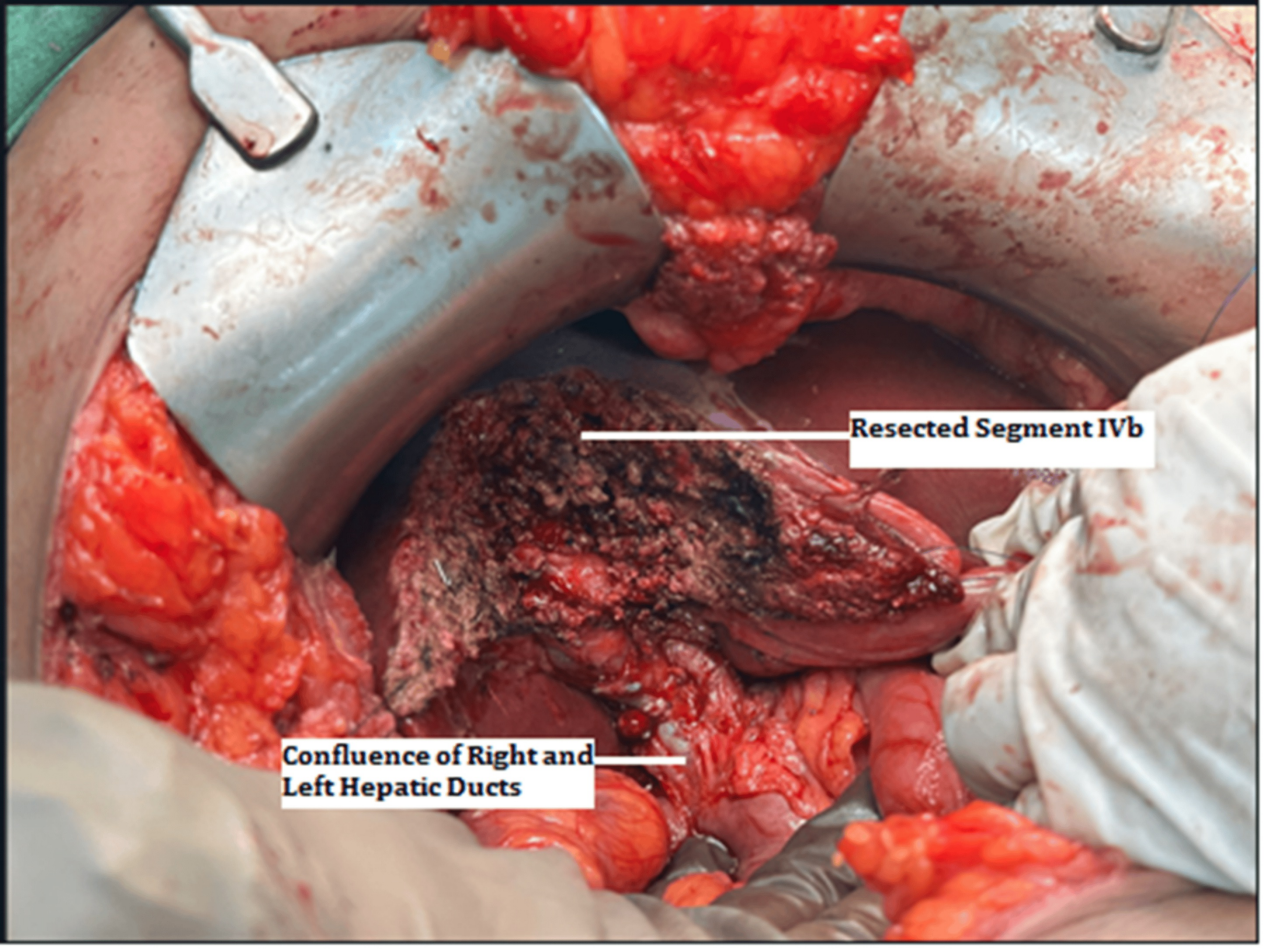
Confluence of hepatic ducts.

With a provisional diagnosis of segment IVb intrahepatic biliary cyst, a decision was taken to resect segment IVb along with the cyst. Under general anaesthesia, Kocher’s incision was made, and cholecystectomy was done. The common hepatic artery was identified, and the right and left hepatic branches were identified, dissected, and looped for proximal control. Intraoperative USG was performed to identify the site of the intrahepatic biliary cyst with calculi, which corresponded to segment IVB of the liver, and USG-guided surface marking was done. The left hepatic arterial and portal vein branches to segment IVB were ligated individually and divided. After resection of segment IVb, the anterior wall of the cyst was seen near the portal confluence (Figure 3) and confirmed by needle aspiration of bile.

**Figure 4:**
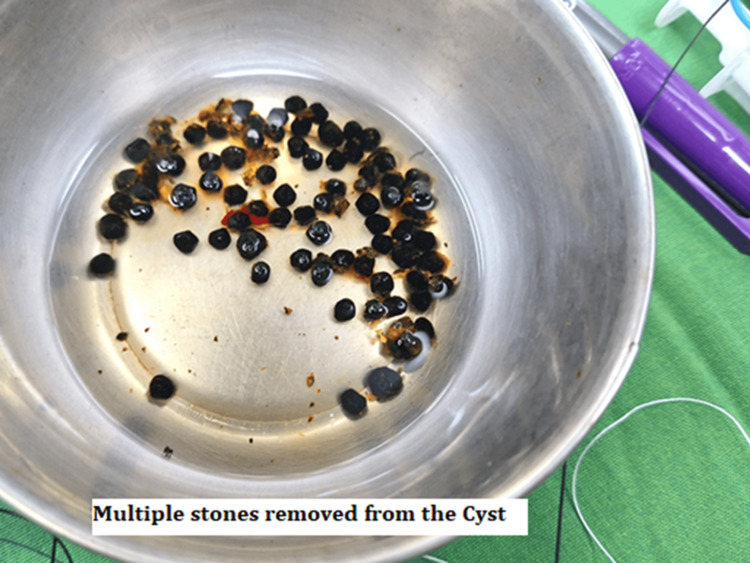
Stones removed from the cyst.

The anterior wall of the cyst was opened transversely, and multiple small stones were removed (Figure 4).

**Figure 5:**
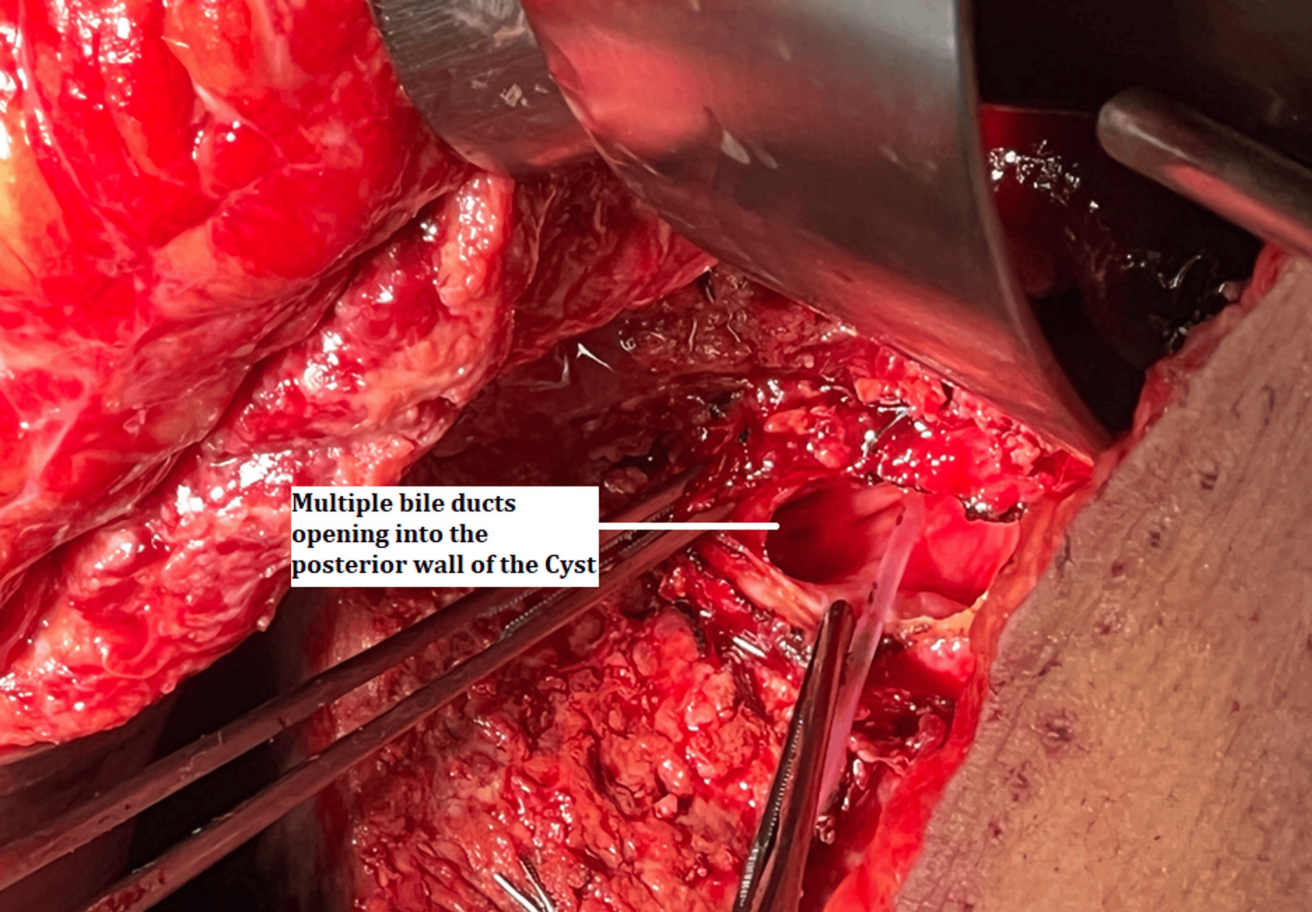
Multiple bile ducts opening into the cyst.

Multiple bile ducts (seven in number) were seen opening into the posterior wall of the cyst (Figure 5).

Partial excision of the anterior wall was done and sent for frozen section to rule out malignancy. The cyst was flushed with saline to flush out all the stones and check the patency of the bile duct. A decision was taken to perform Roux-en-Y hepaticojejunostomy on the remnant of the cyst wall. The postoperative period was uneventful, and the patient was discharged on postoperative day five with no bile leak. She was asymptomatic on the one-week, one-month, and two-month follow-ups.

## Discussion

Biliary tract cysts are cystic duct dilatations of the biliary tree, which may be congenital or acquired and involve extrahepatic and/or intrahepatic segments. The majority of the cases are diagnosed in early childhood, but up to 25% cases are diagnosed in adults, with pain being the most common presenting complaint [1]. Recurrent cholangitis is also a presentation in a few cases because of bacterial colonization caused by bile stasis and stones [4].

Extrahepatic biliary tract cysts were first classified by Alonso-Lej et al. in 1959 [5], and revised by Todani in 1977 to include intrahepatic cysts [3]. According to the original Todani classification, intrahepatic cysts (single or multiple) are described as type V cysts. Multiple intrahepatic cysts without hepatic fibrosis have been named Caroli’s disease [6]. In our case, we encountered a single right hepatic duct cyst, which can be termed as a localized type V cyst. To gain access to the cyst, we had to resect segment 4b of the liver. Surgical options for the resection of this cyst included right hepatectomy (which is excessive) versus partial excision of the cyst and drainage into the Roux limb of a Roux-en-Y hepaticojejunostomy. As the surgical approach to a solitary intrahepatic cyst is different from that of classic Caroli’s disease (Todani type V), we suggest expanding the Todani classification to include solitary intrahepatic cysts as a subtype of type V biliary tract cyst.

A new type of biliary cyst with isolated dilatation of the cystic duct was described by Serena Serradel et al. [7] in 1991, which was termed as type VI choledochal cyst. In 2015, Bhoil et al [8] proposed dividing type VI cysts into the following two types: type VIA with only cystic duct dilatation, and type VIB with combined dilatation of cystic duct and the common bile duct. In 2024, Alghamdi [9] proposed that despite the widespread use of Todani classification, it needs to be updated and improved, considering the numerous reports of uncommon choledochal cyst types and new insights into their etiology. They also proposed that cysts surrounding the confluence of hepatic ducts be termed type VII biliary cysts. Few other cases have been reported with solitary biliary cysts at the confluence of right and left hepatic ducts, out of which one reported choledocholithiasis [10,11].

Although the majority of cases can be classified using the Todani classification system, there have been a few reported cases that do not fit into this classification. These atypical presentations highlight the need for continued evaluation and possible refinement of the existing classification criteria. Hence, we propose expanding the Todani classification to include type VA, VB, VIA, VIB, and VII cysts, as mentioned earlier.

**Figure 6:**
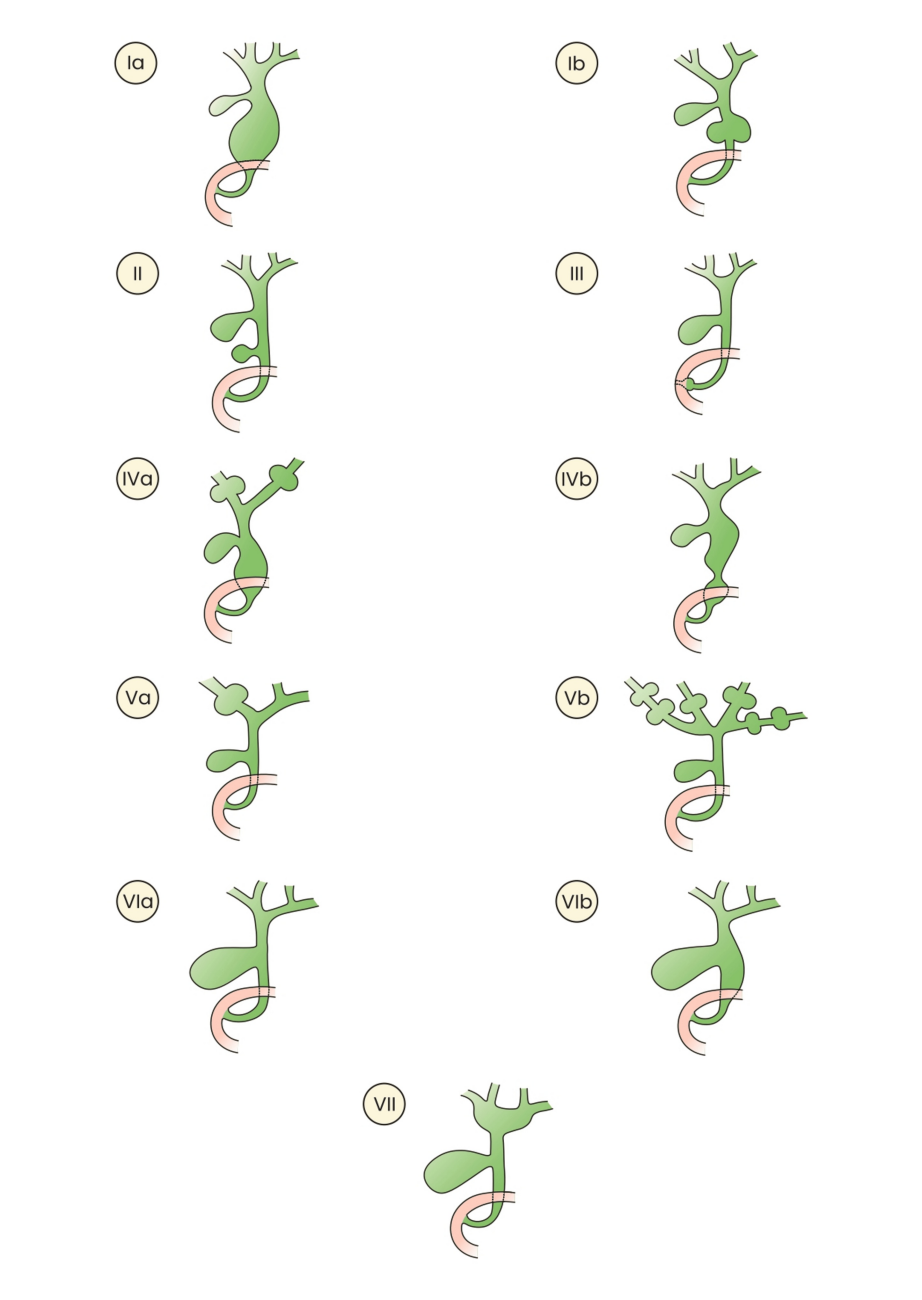
New proposed expanded Todani classification. Source: Authors’ own creation.

The proposed expanded classification is as follows: type 1a: cystic dilatation of the extrahepatic duct; type 1b: focal segmental dilatation of the extrahepatic duct; type 1c: diffuse or fusiform dilatation of the entire extrahepatic duct; type II: diverticulum of the extrahepatic duct; type III: choledochocele (intramural dilatation of the common bile duct within the duodenal wall); type IVA. Multiple cysts at the intra- and extrahepatic ducts; type IVB: multiple cysts at the extrahepatic duct only; type VA: Single intrahepatic bile duct cyst; type VB: multiple intrahepatic bile duct cysts (Caroli’s disease); type VIA: isolated dilatation of the cystic duct; type VIB: combined dilatation of the cystic duct and the common bile duct; type VII: cysts surrounding the confluence of hepatic ducts (Figure 6).

As these cases are rare, we duly acknowledge limitations, such as the single-case nature of the study and the need for further validation of the proposed classification with additional cases.

## Conclusions

Isolated cystic dilatations of intrahepatic bile ducts are extremely uncommon. While the Todani classification system effectively categorizes most choledochal cysts, certain atypical presentations remain unclassified. With the advancement of better imaging modalities over the decades, new rare cases have been reported that do not fit into the original Todani classification of biliary duct cysts. Hence, there is a need for refinement of the existing classification criteria to include these rare cases. Thus, we propose an expansion of the Todani classification to include these uncommon variants as type VA, VB, VIA, VIB, and VII cysts.
